# Van der Waals epitaxial growth of single-crystal molecular film

**DOI:** 10.1093/nsr/nwae358

**Published:** 2024-10-15

**Authors:** Lixin Liu, Penglai Gong, Kailang Liu, Bingrong Huang, Zhihao Zhang, Yingshuang Fu, Yu Wu, Yinghe Zhao, Meihui Wang, Yongshan Xu, Huiqiao Li, Tianyou Zhai

**Affiliations:** State Key Laboratory of Materials Processing and Die & Mould Technology, School of Materials Science and Engineering, Huazhong University of Science and Technology, Wuhan 430074, China; Key Laboratory of Optic-Electronic Information and Materials of Hebei Province, College of Physics Science and Technology, Hebei University, Baoding 071000, China; State Key Laboratory of Materials Processing and Die & Mould Technology, School of Materials Science and Engineering, Huazhong University of Science and Technology, Wuhan 430074, China; State Key Laboratory of Materials Processing and Die & Mould Technology, School of Materials Science and Engineering, Huazhong University of Science and Technology, Wuhan 430074, China; Wuhan National High Magnetic Field Center, School of Physics, Huazhong University of Science and Technology, Wuhan 430074, China; Wuhan National High Magnetic Field Center, School of Physics, Huazhong University of Science and Technology, Wuhan 430074, China; State Key Laboratory of Materials Processing and Die & Mould Technology, School of Materials Science and Engineering, Huazhong University of Science and Technology, Wuhan 430074, China; State Key Laboratory of Materials Processing and Die & Mould Technology, School of Materials Science and Engineering, Huazhong University of Science and Technology, Wuhan 430074, China; State Key Laboratory of Materials Processing and Die & Mould Technology, School of Materials Science and Engineering, Huazhong University of Science and Technology, Wuhan 430074, China; State Key Laboratory of Materials Processing and Die & Mould Technology, School of Materials Science and Engineering, Huazhong University of Science and Technology, Wuhan 430074, China; State Key Laboratory of Materials Processing and Die & Mould Technology, School of Materials Science and Engineering, Huazhong University of Science and Technology, Wuhan 430074, China; State Key Laboratory of Materials Processing and Die & Mould Technology, School of Materials Science and Engineering, Huazhong University of Science and Technology, Wuhan 430074, China

**Keywords:** van der Waals epitaxy, inorganic molecular crystal, layer-by-layer growth, single-crystal film, dielectric

## Abstract

Epitaxy is the cornerstone of semiconductor technology, enabling the fabrication of single-crystal film. Recent advancements in van der Waals (vdW) epitaxy have opened new avenues for producing wafer-scale single-crystal 2D atomic crystals. However, when it comes to molecular crystals, the overall weak vdW force means that it is a significant challenge for small molecules to form a well-ordered structure during epitaxy. Here we demonstrate that the vdW epitaxy of Sb_2_O_3_ molecular crystal, where the whole growth process is governed by vdW interactions, can be precisely controlled. The nucleation is deterministically modulated by epilayer–substrate interactions and unidirectional nuclei are realized through designing the lattice and symmetry matching between epilayer and substrate. Moreover, the growth and coalescence of nuclei as well as the layer-by-layer growth mode are kinetically realized via tackling the Schwoebel-Ehrlich barrier. Such precise control of vdW epitaxy enables the growth of single-crystal Sb_2_O_3_ molecular film with desirable thickness. Using the ultrathin highly oriented Sb_2_O_3_ film as a gate dielectric, we fabricated MoS_2_-based field-effect transistors that exhibit superior device performance. The results substantiate the viability of precisely managing molecule alignment in vdW epitaxy, paving the way for large-scale synthesis of single-crystal 2D molecular crystals.

## Teaser

This paper reports the van der Waals epitaxial growth of single-crystal Sb_2_O_3_, paving the way for precisely managing molecule alignment over nucleation and growth kinetics.

## INTRODUCTION

Single-crystal epitaxy technology, which bridges the gap between advanced materials science and integrated device manufacturing, plays a crucial role in microelectronics and optoelectronics applications. Recent investigations confirmed that van der Waals (vdW) epitaxy is a viable strategy for synthesizing large-area, single-crystal 2D layered materials [1–5]. During the epitaxy process, adatoms attach to the edges of oriented nuclei with in-plane chemical bonding, aligning into a long-range ordered structure and eventually seamlessly stitching into 2D single-crystal film [6–8].

Despite the significant progress in vdW epitaxial growth of atomic crystals with layered structure, the synthesis of large-area single-crystal molecular film through this technique has not yet been reported. Molecular crystals are composed of individual molecules that interact through vdW force, structurally distinguishing them from atomic crystals. Although the unique structure of molecular crystals bestows upon them fascinating (opto)electronic properties, it simultaneously brings challenges in attaining a long-range ordered structure during epitaxy, as both the out-of-plane and in-plane growth are governed by weak vdW force in place of strong chemical bonds [9].

Here, utilizing the substrate-guided strategy, we develop a vdW epitaxy technique to synthesize 2D single-crystal molecular film. The unidirectional Sb_2_O_3_ triangular domains are modulated through the lattice and symmetry matching between molecular epilayer and substrate. Furthermore, the layer-by-layer growth mode of Sb_2_O_3_ molecules is realized by precisely controlling the kinetic process, which enables the fabrication of uniform, dense and single-crystal molecular film with desirable thickness. Incorporating a highly oriented Sb_2_O_3_ film on graphene as the gate dielectric in MoS_2_-based field-effect transistors (FETs) results in an ultralow subthreshold swing (SS) that approaches the thermionic limit of 60 mV dec^−1^. Additionally, in contrast to FETs using polycrystalline Sb_2_O_3_ dielectric film of the same thickness (5 nm), there is a 4-orders-of-magnitude reduction in gate leakage current.

## CONTROLLED vdW EPITAXIAL GROWTH OF MOLECULAR CRYSTALS

**Figure 1.**
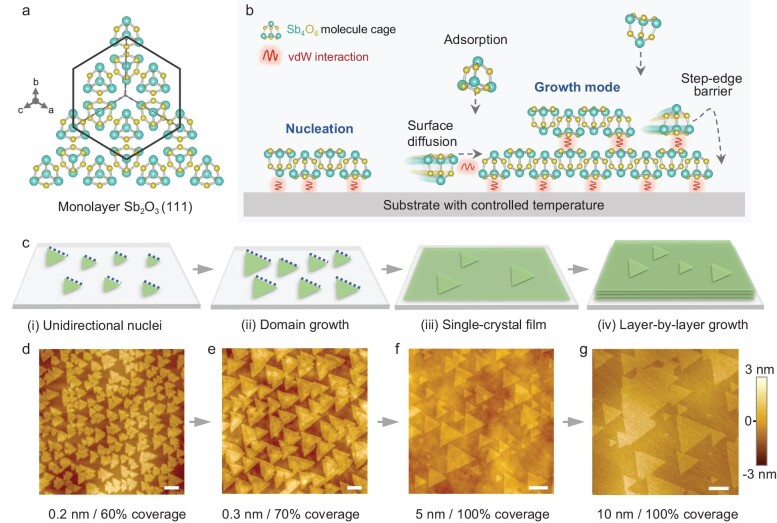
Controlled growth for single-crystal Sb_2_O_3_ film. (a) Schematic of monolayer Sb_2_O_3_ with (111) plane. (b) Schematic illustration of growth modes and molecule behavior during the typical vdW epitaxy process. (c) Illustration of the proposed growth pathway of single-crystal Sb_2_O_3_ thin film. The substrate guides the unidirectional arrangement of nuclei at the nucleation stage, then the domains grow and seamlessly stitch into a film, followed by the film thickening through the layer-by-layer growth mode. (d–g) Corresponding atomic force microscopy (AFM) images of single-crystal Sb_2_O_3_ domains/films grown at different stages. Scale bars, 200 nm.

As shown in Fig. 1a, within the face-centered cubic cell structure of the Sb_2_O_3_ molecular crystal, the lattice sites are filled with Sb_4_O_6_ molecular cages [10]. vdW interaction is the exclusive force between Sb_4_O_6_ cages, which determines the arrangement of molecules [11–14]. Figure 1b illustrates the assembly process of small molecules, following the classic nucleation-and-growth theory [15,16]. However, the absence of strong chemical bonding results in an uncontrolled epitaxial process [17], including disordered molecular arrangement, stochastic nucleation orientation and unfavorable islanding growth mode. While previous studies have reported the synthesis of 2D materials/Sb_2_O_3_ heterostructure flakes [18–20], a comprehensive analysis on nucleation orientation and growth mode are still lacking, both of which are vital for the development of high-quality molecular films. Addressing the inherent challenges for vdW epitaxy of molecular crystals necessitates meticulous thermodynamic and kinetic regulations on the nucleation, coalescence and growth stages, which will be thoroughly examined in the subsequent sections. Considering the weak in-plane molecular interaction, the coupling effect between substrate and molecular epilayer is essential for guiding the nucleation orientation [1]. In the growth mode, the substrate temperature has a direct impact on the step-edge barrier, which in turn determines the interlayer diffusion [21].

There are four typical stages during the precisely controlled vdW epitaxy process (Fig. 1c). At the nucleation stage, the discrete molecules are aligned into unidirectional nuclei along the lattice matching direction with the lowest formation energy (Fig. 1d), then the size of Sb_2_O_3_ nuclei increases, fed by the molecules diffusing on the surface (Fig. 1e). The single-crystal Sb_2_O_3_ films were obtained after the full coalescence of unidirectional domains (Fig. 1f), and the thickness of the film could be precisely controlled by the modulated layer-by-layer growth mode (Fig. 1g). In the following, we will respectively demonstrate our strategies to achieve such control in different stages of the deposition process.

## UNIDIRECTIONAL NUCLEATION OF SINGLE-CRYSTAL Sb_2_O_3_ DOMAINS

**Figure 2.**
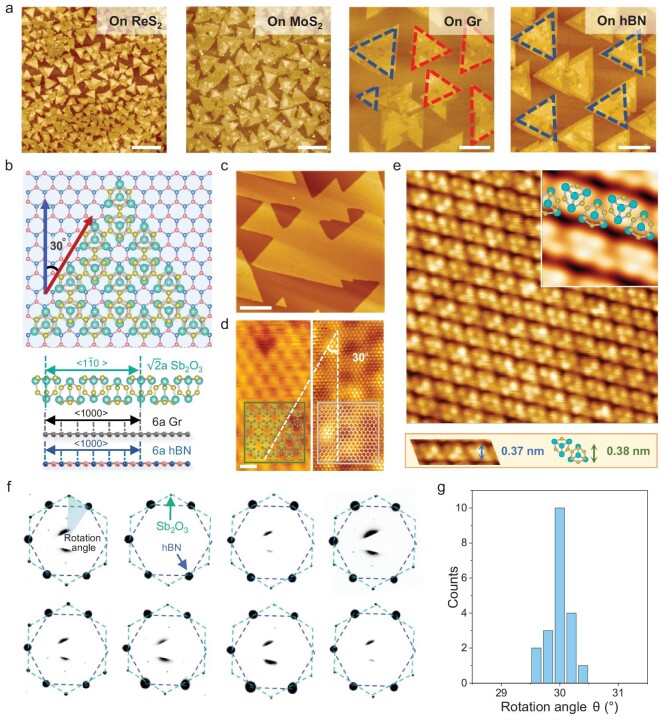
vdW epitaxy of triangular Sb_2_O_3_ domains. (a) AFM images of Sb_2_O_3_ domains grown on ReS_2_, MoS_2_, graphene and hBN substrate, respectively. Scale bars, 200 nm. (b) Schematic illustration of the lattice matching relationship between Sb_2_O_3_, graphene and hBN. (c) STM image of highly oriented Sb_2_O_3_ domains grown on graphene. Scale bar, 50 nm. (d) Zoomed lattice-resolved STM images of Sb_2_O_3_ and the adjacent graphene substrate, revealing a misorientation angle of 30°. Scale bar, 1 nm. (e) Atomic-resolved STM image of Sb_2_O_3_. (f) SAED patterns after color inversion processing captured from eight random positions of Sb_2_O_3_ domains grown on hBN. (g) Histogram of the orientation distribution from 20 SAED patterns.

Sb_2_O_3_ molecules are deposited onto a desired substrate through standard thermal evaporation with the intact molecular cage structure preserved [22,23] (Fig. S1). Guiding molecular assembly for single-crystal growth hinges on achieving unidirectional nucleation, with the interaction between substrate and molecules being pivotal. In the vdW epitaxy of Sb_2_O_3_, the layered materials with dangling-bond-free surfaces were used as the substrate, on which the deposited molecules enjoyed a high surface diffusivity and a thermodynamic equilibrium nucleation process [24]. The formation of 2D material domains can be divided into two processes, including the diffusion of precursors on substrate and their attachment to active nuclei edges for aggregation [25]. The formation of polygonal domains through nucleation requires fast diffusion of ad-molecules, while their shape is determined by the crystalline structure, following an energetically favorable arrangement [26]. The substrate temperature determines the average kinetic energy of molecules. The shape of Sb_2_O_3_ nuclei on 2D substrates remained dendritic at 20°C (Fig. S2), irrespective of the substrate variations. However, a significant alteration in nuclei shape occurs with an increase in substrate temperature by dozens of degrees. When the substrate temperature is elevated to 50°C, the Sb_2_O_3_ molecules assemble into triangular nuclei on a series of 2D substrates (Fig. 2a). In contrast, due to the pinning effect of dangling bonds, granular nuclei are persistently formed on a 3D dielectric substrate (like SiO_2_) regardless of the change in substrate temperature (Fig. S3).

It is noteworthy that the orientations of Sb_2_O_3_ nuclei depend on the crystalline structure of substrates. The orientation of an Sb_2_O_3_ nucleus seemed random on ReS_2_ and MoS_2_ substrates but became highly oriented on graphene and hBN substrates. Systematic crystal structure characterizations were carried out. We performed scanning tunneling microscopy (STM) on the Sb_2_O_3_ domains grown on the bilayer graphene substrate. The highly oriented triangular Sb_2_O_3_ domains can be observed in Fig. 2c, and the relative rotation angle between epilayer and substrate was confirmed as 30° in the zoomed lattice image (Fig. 2d). In the atomic-resolution image (Fig. 2e), Sb_4_O_6_ molecular cages with (111) exposed surfaces were distinguished, and the lattice spacing was measured as 0.37 nm, in agreement with the ideal molecular cage structure [27].

The schematic of atomic structure shows clear evidence of an epitaxial relationship with lattice matching. The configuration of Sb_2_O_3_ (111) plane on hBN or graphene (0001) plane with a misorientation of 30° is shown in Fig. 2b. From the side view along the zigzag direction of the substrate (parallel to < 1–10 > direction of Sb_2_O_3_), we can see that the supercell of Sb_2_O_3_ ($\sqrt {{\mathrm{2}}\ } $ × 11.15 Å = 15.77 Å) matches the 6 × 6 supercell of hBN (6 × 2.51 Å = 15.06 Å) and graphene (6 × 2.46 Å = 14.76 Å). Consequently, the lattice mismatches are extracted as 4.5% for hBN and 6.4% for graphene, while they are estimated, in clear contrast, to be over 20% for MoS_2_ and ReS_2_.

The well-defined epitaxial relationship was further proven by the statistical analysis of selective area electron diffraction (SAED) patterns in transmission electron microscopy (TEM) characterization. We collected the SAED patterns from different regions of unidirectional Sb_2_O_3_ domains grown on hBN. The original images are shown in Fig. S4, while Fig. 2f exhibits the patterns after color inversion processing for enhanced contrast, all patterns demonstrating identical rotation relationship. The two sets of diffraction dots can be assigned to the (111) plane of Sb_2_O_3_ and (0001) plane of hBN, respectively, and the angles between them are measured as 30 ± 0.5° (Fig. 2g).

**Figure 3.**
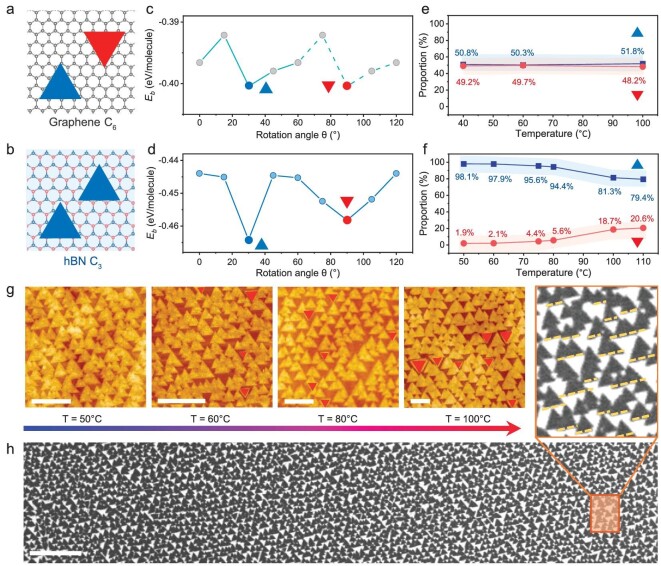
Mechanism of unidirectional nucleation. (a and b) Schematic illustration of Sb_2_O_3_ orientations grown on graphene with C_6_ lattice symmetry (a) and hBN with C_3_ lattice symmetry (b). (c and d) The calculated binding energies (*E*_b_) of Sb_2_O_3_ domains as a function of the rotation angle (θ) on graphene (c), and on hBN (d). (e and f) The statistical distribution of Sb_2_O_3_ unidirectional nucleation proportion at different temperatures on graphene (e) and hBN (f). (g) AFM images of Sb_2_O_3_ domains grown on hBN at temperatures of 50°C to 100°C. Scale bars, 200 nm. (h) SEM image of Sb_2_O_3_ domains grown on hBN at 75°C, indicating the unidirectional domains at the large scale. Scale bar, 2 μm.

We also performed density functional theory (DFT) calculations to investigate the orientation relationship between the Sb_2_O_3_ epilayer and substrate. The orientation of the epilayer is influenced not only by lattice matching with the substrate but also by the symmetry of the substrate [28]. As illustrated in Fig. 3a and b, considering that the (111) plane of the Sb_2_O_3_ possesses a 3-fold rotation symmetry, the domains should have two different degenerate directions on a C_6_ symmetric substrate and only one direction on the substrate with lower C_3_ symmetry [29], which is consistent with the observed result in Fig. 2a and b. Note that our experiments demonstrated that Sb_2_O_3_ domains nucleate directly at the inherent vdW surface of the substrate rather than the step edges or defects region in substrate (Fig. S5). Hence, we established graphene and hBN supercells as the substrates and placed a triangular ‘crystal nucleus’ composed of nine Sb_2_O_3_ molecules over them, as the calculation configurations. We constructed a series of highly symmetrical structures under various relative rotation angles (Figs S6 and S7), and for each orientation, the minimum binding energies were extracted and plotted in Fig. 3c and d. The smaller binding energy indicates a more stable system. Our results show that the binding energy varies periodically versus orientation angle. On the substrate of graphene, the binding energy shows local minima at both 30° and 90°, indicating that the parallel and the antiparallel orientation are identically stable states for nucleation. In the statistical analysis of the domain orientation, these two antiparallel orientations appear with nearly equal probability regardless of the change in growth temperature (Fig. 3e, Fig. S8 and Table S1). However, the lower symmetry of the hBN substrate breaks the energetic degeneracy, offering a unique minimum at a rotation angle of 30°. The energy difference between two antiparallel rotation angles results in the unidirectional alignment of Sb_2_O_3_ domains. Quantitatively, the energy difference (ΔE_b_) reaches 0.11 eV assuming the critical nucleus is composed of 18 Sb_2_O_3_ molecules (with a side length of 3 nm), and the thermodynamic probability can be estimated by 1/(1 + exp($\frac{{{\mathrm{\Delta }}{{{\mathrm{E}}}_{\mathrm{b}}}}}{{{{{\mathrm{K}}}_{\mathrm{b}}}{\mathrm{T}}}}$)) [30]. In this way, the unidirectional alignment proportion was calculated as being >98% at T = 50°C, in excellent agreement with the statistical result of thousands of domains (Table S2). Given that temperature affects the alignment proportion exponentially, the proportion dropped below 95% with a dozens-of-degree (Fig. 3f) increase in temperature. The domains with antiparallel orientation became increasingly prevalent as the growth temperature continued to rise, as shown in Fig. 3g.

For a better understanding of the epilayer–substrate interaction mechanism, we decomposed the binding energy into the contribution of vdW energy and other energies (mainly electrostatic energy). The results revealed that vdW interaction contributes >93% to the binding energy, that is to say, the other factors can be ignored (Fig. S9), indicating that the vdW interaction between unidirectional Sb_2_O_3_ seeds and the 2D substrate is effective enough to guide the nucleation orientation. In addition, upon reaching a fully relaxed state, there are significant vdW gaps with an average equilibrium distance of ∼3 Å across all configurations with diverse rotation angles (Figs S10 and S11), further confirming that vdW force is the exclusive interaction. What we discussed above unambiguously shows that the key to obtaining unidirectional aligned domains in vdW epitaxial growth of molecular crystals lies in controlling the interplay between epilayer and substrates, which is dominated by lattice matching and the structure symmetry relationship between them. We characterized >2500 triangular Sb_2_O_3_ domains over a much larger scale under scanning electron microscopy (SEM), all of which showed preferred orientation in the visual field (Fig. 3h).

## KINETIC CONTROL OF GROWTH MODE

**Figure 4.**
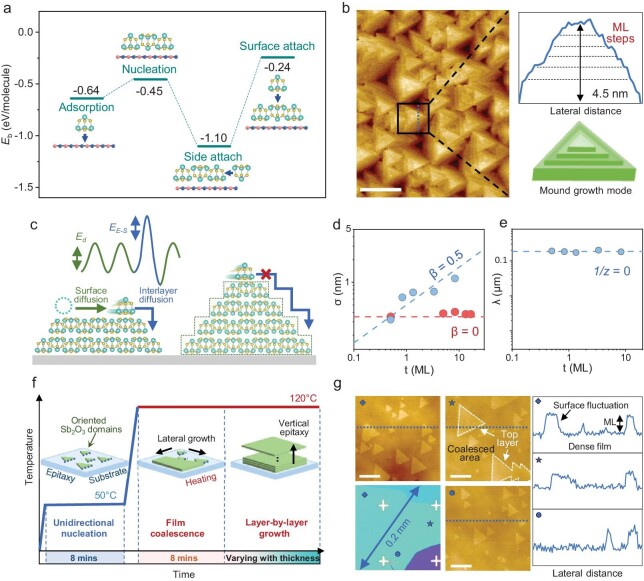
Kinetically controlled layer-by-layer growth mode. (a) The representative structures and corresponding calculated binding energy in the growth process of Sb_2_O_3_ on hBN. (b) AFM image of Sb_2_O_3_ morphology grown at 50°C, and corresponding profile line and schematic of the Sb_2_O_3_ mound. Scale bar, 200 nm. (c) Illustration of the energy barrier of an Sb_2_O_3_ molecule for interlayer and intralayer mass transport. (d and e) Evolution of the roughness (d) and average mound separation (e) with film thickness, in which the blue dots represent the growth carried out at 50°C, and the red dots represent 100°C. (f) Evolution of the growth temperature with different growing stages to achieve unidirectional nucleation and layer-by-layer growth mode. (g) AFM and optical images of a dense and flat 5 nm Sb_2_O_3_ film grown on hBN with a side length over 0.2 mm, and the corresponding profile line. The different symbols denote the capture locations. Scale bars, 200 nm.

As well as controlling the initial nucleation and coalescence of nuclei, the layer-by-layer growth mode is also required to grow a flat single-crystal film of desirable thickness. To investigate the growth mode of Sb_2_O_3_, we first analyze the attachment behaviors of a deposited Sb_2_O_3_ molecule. We carried out DFT calculations to investigate the preferential attachment position, taking four different configurations into consideration (Fig. 4a). The results clearly indicate that the deposited molecules tend to nucleate on hBN and the subsequent adsorbed molecules energetically prefer to attach to the side of the nucleus rather than on the top. The layer-by-layer growth mode is thus thermodynamically favored in the epitaxy of Sb_2_O_3_. However, on the contrary, we observe an apparent islanding growth in our experiments. Continuous deposition of Sb_2_O_3_ on hBN substrate at 50°C leads to the formation of mounds instead of a flat film, and monolayer steps can be identified from the sides of the mounds (Fig. 4b).

It is evident that thermodynamics does not dictate the mounded growth mode, thus we investigated its growth kinetics. Figure 4c demonstrates the molecular diffusion pathway and corresponding change in energy. Molecules diffuse along the surface of nuclei with a diffusion barrier (E_d_) [31]. When diffusing down the edge, the molecules are required to overcome an additional Ehrlich-Schwoebel barrier (E_E-S_), which determines the interlayer mass transport [32,33]. The kinetic energy of molecules was not enough to cross the E_E-S_ at 50°C, and only in-plane diffusion was allowed, shaping the mound-like surface morphology. The quantitative research on roughness (σ) and average separation (λ) of mounds further confirmed the existence of E_E-S_ [34]. The blue dashed line in Fig. 4d shows that the roughness σ evolved with the square root of thickness ($\sqrt {\mathrm{t}} $). Accordingly, the growth exponent (β) was evaluated as 0.5 through the power-law fit, which is in good agreement with the predicted mounded growth mode [35]. Furthermore, by estimating the function between the average mound separation (λ) and thickness (t), a dynamic growth exponent (1/*z*) was extracted as 0 (Fig. 4e), indicating that the separation distance remains unchanged regardless of the increase in thickness and the mass transport between individual mounds is deeply suppressed [34,36]. Such analysis points to a typical islanding growth mode induced by the Ehrlich-Schwoebel barrier at the step edges. We can therefore conclude that the growth mode can be kinetically modulated by tackling the step-edge energy barrier.

Since the optimal unidirectional nucleation ratio is achieved at a moderate temperature, while enhanced interlayer mass transport requires a higher temperature, we designed the deposition process to independently control the nucleation and growth stages. The programed growing stages are shown in Fig. 4f. The unidirectional nucleation was carried out at 50°C with a thickness of 1 nm. Deposition rate was set at 0.02 Å/s, resulting in a nucleation time of ∼8 mins. Subsequently, the substrate was heated to 120°C to boost the interlayer diffusion and film coalescence with the same deposition rate and duration (0.02 Å/s, 8 mins). In such a way, a layer-by-layer growth mode is kinetically realized, allowing for precise regulation of the thickness of the Sb_2_O_3_ single-crystal film and maintaining a flat surface regardless of increase in thickness. To better understand the effect of temperature on nucleation and growth stage, we compared films synthesized under different conditions. The results unambiguously showed that the films with uniform morphology and unidirectional domains appeared only with low nucleation temperature and high growth temperature, in line with the aforementioned mechanism (Fig. S12).

A high-quality single-crystal Sb_2_O_3_ film with thickness of 5 nm was synthesized through this multi-stage growth strategy, which remains dense and homogenous over the whole hBN substrate, with a lateral size of 0.2 mm (Fig. 4g). The whole growing process can be seen in quasi-*in-situ* SEM images (Fig. S13), and Sb_2_O_3_ films with different thicknesses can be precisely synthesized (Fig. S14). Moreover, we investigated the surface characteristics of the as-grown single-crystalline Sb_2_O_3_ film and performed a quantitative analysis of its orientation and thickness distribution. As depicted in Fig. S15, the unidirectional alignment reached 98.24%, and most of the film surface undulations remain within the range of a single molecular layer thickness.

## HIGHLY ORIENTED Sb_2_O_3_ FILMS AS A GATE DIELECTRIC IN 2D FETs

The precise control enables the oriented nucleation, coalescence and layer-by-layer growth of Sb_2_O_3_ on graphene and hBN substrates. We demonstrated that the high-quality Sb_2_O_3_ film can serve directly as an excellent gate dielectric in 2D FETs. Here we chose few-layer graphene as the substrate, which then functions as a back-gate electrode of the FET, allowing the deposition of the highly oriented Sb_2_O_3_ layer as the exclusive gate dielectric.

**Figure 5.**
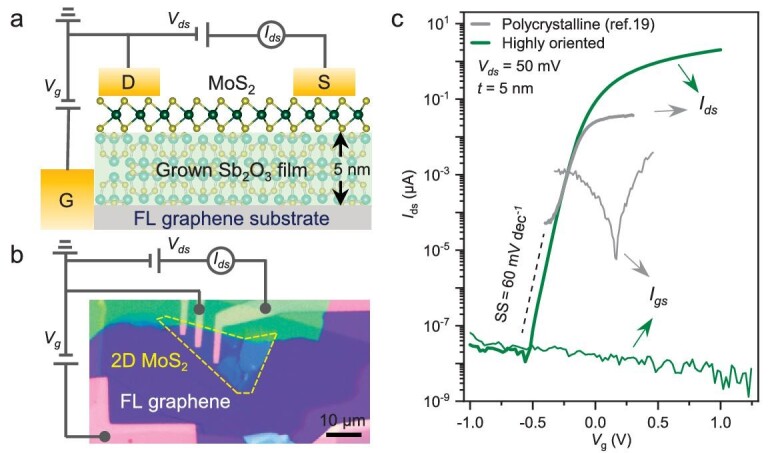
An FET based on the highly oriented Sb_2_O_3_/graphene film. (a) Schematic of a MoS_2_ FET using highly oriented Sb_2_O_3_ thin film as the dielectric and underlying graphene as the gate electrode, respectively. (b) Optical image of the FET device. (c) Transfer characteristic curve (*I*_ds_-*V*_g_) of the FET at room temperature.

We fabricated 2D FETs by transferring MoS_2_ flakes as the channel and depositing the electrodes (Fig. 5a and b, see Experimental Section in Supplementary Data for details). The thickness of the Sb_2_O_3_ dielectric film was set at 5 nm, which corresponds to an equivalent oxide thickness (EOT) of 1.6 nm. Figure 5c illustrates the transfer characteristics of the back-gated FET using highly oriented Sb_2_O_3_ film as the dielectric layer, exhibiting a source-to-drain current (I_ds_) switching over 10^7^ within a low gate voltage of 0.5 V. Such high gating efficiency and low leakage current (I_gs_) compared with FETs based on dielectrics of polycrystalline [22] or rough Sb_2_O_3_ film (Fig. S16) can obviously be attributed to the improved crystal quality with deeply reduced grain boundaries and homogeneous thickness. Furthermore, the atomically flat and dangling-bond-free vdW interface between the oriented Sb_2_O_3_ and MoS_2_ endows the FET with an ultralow SS value approaching the thermionic limit of 60 mV dec^−1^. The suppressed interfacial states are also confirmed by the negligible small hysteresis window [37] in the double-sweep transfer curves (Fig. S17). Compared to previously reported dielectric layers [22,38–40] (Table S3), highly oriented Sb_2_O_3_ exhibits superior quality, ideal vdW interfaces and excellent gating efficiency, making it a promising candidate for scaled-down 2D devices. We also demonstrated that the highly oriented Sb_2_O_3_ film is uniform on the centimeter scale (Figs S18 and S19), which is promising in terms of fabricating the array devices.

Besides serving as FETs that are directly gated by applying voltage at graphene, the devices supported on SiO_2_/Si substrate can potentially work as floating-gate field-effect transistors (FGFETs) [41]. In the FGFET, degenerately doped Si serves as the bottom gate, and the graphene works as the floating gate, which is separated from the MoS_2_ channel by a 5-nm-thick highly oriented Sb_2_O_3_ tunneling layer. The SiO_2_ functions as the blocking layer beneath the graphene. The basic characterization of the FGFET was performed by double sweeping the bottom gate within ±6 V, and an obvious memory window of 2.9 V was demonstrated (Fig. S20). We only verified the feasibility of the device, and more in-depth investigations are required to realize the complex programmable logic operations.

Provided we have suitable large-scale substrates, wafer-scale single-crystal Sb_2_O_3_ can potentially be grown via our controlled vdW epitaxy. Recent studies have reported the synthesis of wafer-scale single-crystal hBN monolayers [28,42], which offer a solid foundation from which to grow single-crystal wafer-scale Sb_2_O_3_ film in the future. The thickness of the dielectric layer can be facilely controlled, which effectively mitigates the current leakage issues associated with thin hBN film.

## CONCLUSION

We systematically studied the thermodynamics and kinetics in vdW epitaxy of molecular crystals and demonstrated the growth of single-crystal film via precise control over the nucleation and growth processes. Moreover, a layer-by-layer growth mode was achieved in vdW epitaxy by kinetically tackling the step-edge energy barrier. Based on the precise control of thermodynamics and kinetics, single-crystal Sb_2_O_3_ molecular film with a desirable thickness was produced. The high-quality Sb_2_O_3_ films grown on graphene were used as the gate stack, and enabled the high performance of the FET. We provide an exhaustive explanation of the underlying mechanism of nucleation and growth in vdW epitaxy of molecular crystals, which creates unprecedented opportunities for the synthesis of large-scale single-crystal molecular crystals.

## Supplementary Material

nwae358_Supplemental_File
