# Supplementary material for: Van der Waals epitaxial growth of single-crystal molecular film
Source: Natl Sci Rev. 2024 Oct 15;11(11):nwae358. doi: 10.1093/nsr/nwae358 (PMC11556342; doi:10.1093/nsr/nwae358)
Supplement: nwae358_Supplemental_File [file nwae358_supplemental_file.pdf]

Supporting Information

Van der Waals epitaxial growth of single-crystal molecular film

Lixin Liu[1,†], Penglai Gong[2,†], Kailang Liu[1], Bingrong Huang[1], Zhihao Zhang[3], Yingshuang Fu[3], Yu Wu[1], Yinghe Zhao[1], Meihui Wang[1], Yongshan Xu[1], Huiqiao Li[1] and Tianyou Zhai[1,*]

[1]State Key Laboratory of Materials Processing and Die & Mould Technology, School of Materials Science and Engineering, Huazhong University of Science and Technology, Wuhan 430074, China;
[2]Key Laboratory of Optic-Electronic Information and Materials of Hebei Province, College of Physics Science and Technology, Hebei University, Baoding 071000, China;
[3]Wuhan National High Magnetic Field Center, School of Physics, Huazhong University of Science and Technology, Wuhan 430074, China
***Corresponding author.** E-mail: zhaity@hust.edu.cn
[†]Equally contributed to this work.

1. Experimental Section

1.1 Sb_2O_3 synthesis

The Sb_2O_3 film were synthesized through a standard thermal evaporation deposition with a standard thermal evaporation system (ZHDS400, Technol) embedded inside a glove box. Sb_2O_3 powder with diameter of 10 μm was used as the evaporation source. (99.999%, Zhongsheng Hengan). The deposition was launched when the base pressure was below 10^{-6} torr, and substrate temperature was stabilized at the setpoint (20°C-120°C). The evaporation rate was precisely controlled to be 0.01-0.02 Å/s by an in-situ crystal quartz monitor, the extremely low deposition rate helped to reach the equilibrium state of Sb_2O_3 growth and maintain the flatness of Sb_2O_3 film. The thickness of the film was monitored, and the deposition will be stopped when the target thickness was reached. Before taking out the samples, the substrates were natural cooled down to room temperature.

1.2 Substrate preparation

Micrometer-scale machinal exfoliated graphene and hBN was used as the substrate. For maintaining the ultraclean surface of 2D substrate, the exfoliation was carried out in the glove box and then transferred into the chamber for Sb_2O_3 deposition. The centimeter-scale single-crystal graphene was synthesized through chemical vapor deposition on a Cu foil.

1.3 Morphological and structural characterization

Optical measurements. The optical images were obtained through an optical microscopy (OLYMPUS, BX51). The morphology and thickness of the Sb_2O_3 film are characterized through an AFM (Bruker, Dimension Icon). The crystalline phase and quality were examined through a confocal Raman spectroscopy (WITec Alpha 300 Raman) with a 532 nm excitation laser at 10 mW. The high laser energy was used to collect the evident peaks signal.

Structural characterization. The growth process of Sb_2O_3 on graphene and hBN were observed through a SEM (FEI Qusanta650 FEG). The as-grown Sb_2O_3/graphene and

Sb_2O_3/hBN film were transferred onto the TEM grid through a PMMA-assisted wet transfer method, and then the SAED patterns are captured through FEI Tecnai G2 F30 operated at 300 kV. For STM measurements, the Sb_2O_3 was deposited onto a bilayer graphene which formed on SiC (0001) substrate after annealing, and the samples were in-situ observed through a custom- made cryogenic Unisoku STM (1200) system at 78 K. Normal W tips were cleaned by e-beam heating and had been characterized on Ag (111) multilayer film before measurements. The conductance spectra were recorded by lock-in detection of the tunneling current with a modulation voltage at 983 Hz feeding into the sample bias.

1.4 Theoretical calculation

The first-principles calculations are carried out within the framework of the density functional theory (DFT) implemented in the PWmat package[1,2]. The norm-conserving pseudopotentials (NCPP)[3] with a plane-wave cutoff of 500 eV is adopted. The generalized gradient approximation (GGA) with Perdew-Burke Ernzerhof (PBE) functional[4] is used to describe the exchange-correlation interaction. A semi-empirical correction scheme of the DFT-D3 method[5] is employed to deal with the van der Waals interaction between layers. Periodic slabs were separated by a vacuum layer of 15 Å in the z-direction to avoid mirror interactions. A k-mesh interpolation less than $0.03\times2\pi$/Å following the Monkhorst-Pack scheme[6] is adopted for sampling the Brillouin zone. The atomic positions are fully relaxed until the maximum force on each atom is less than 0.01 eV/Å.

1.5 Device fabrication and test

For the FET fabrication, a two-dimensional MoS_2 flake was exfoliated on polydimethylsiloxane (PDMS) and transferred onto a 5nm-Sb_2O_3/graphene film as the channel. The electrode patterns were defined by electron beam lithography (EBL, FEI Quanta 650 SEM, and Raith Elphy Plus). A pad was connected to the underlying graphene as the gate electrode. The source and drain electrodes were defined above MoS_2, and Bi/Au contact was used to form low resistance. The FET characteristics were

measured with a semiconductor device analyzer (Keithley 4200) at 300 K, and the chamber underwent a process of pumping to create a high vacuum state, preventing gas adsorption on the surface of devices.

1.6 EOT calculation

EOT allows for a direct comparison of the electrical thickness of the Sb_2O_3 film to the traditional SiO_2, and can be calculated as

$$\mathrm{EOT} = \frac{\varepsilon_{\mathrm{SiO_2}}}{\varepsilon_{\mathrm{Sb_2O_3}}} \cdot t_{\mathrm{Sb_2O_3}}$$

where the $\varepsilon_{\mathrm{SiO_2}}$ is the dielectric constant of SiO_2, typically 3.9, and the value $\varepsilon_{\mathrm{Sb_2O_3}}$ used is 11.5. Substituting 5 nm into the $t_{\mathrm{Sb_2O_3}}$ gives the EOT is 1.6 nm.

2. Supplementary Figures

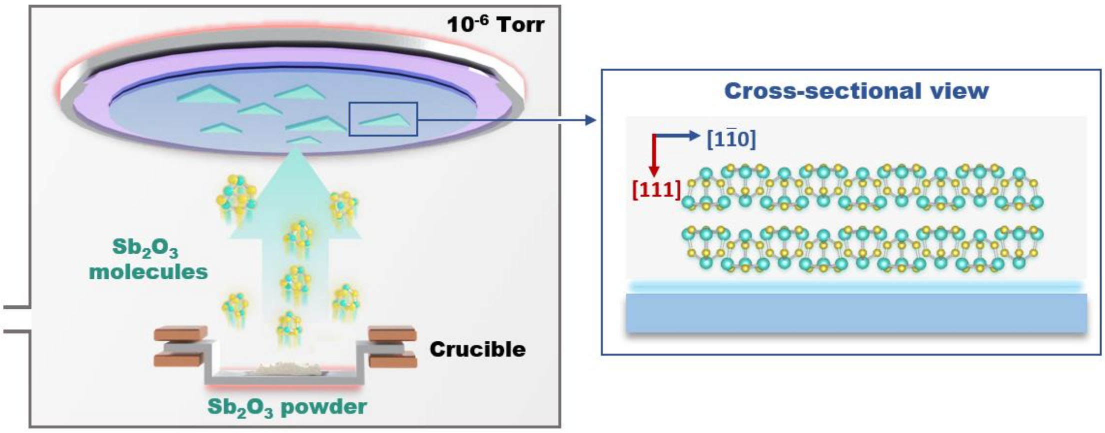

Figure S1. Schematic of preparing Sb_2O_3 thin films through vacuum evaporation. The inset is the cross-sectional view of the as-grown Sb_2O_3.

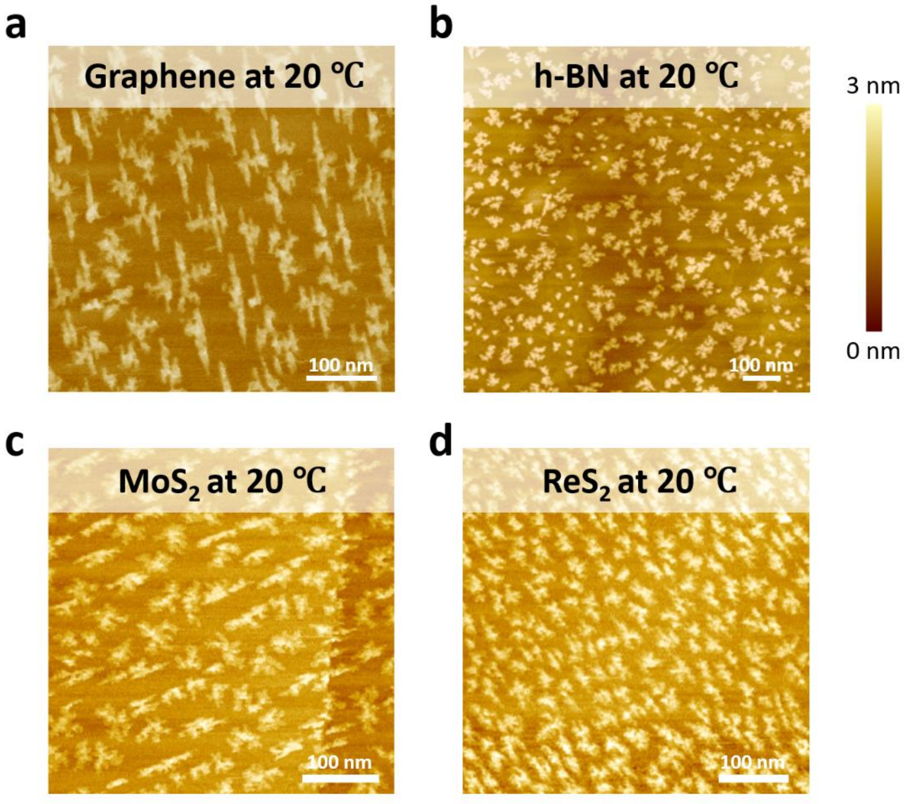

Figure S2. AFM images of Sb_2O_3 grown on different 2D substrates at room temperature (20 ℃).

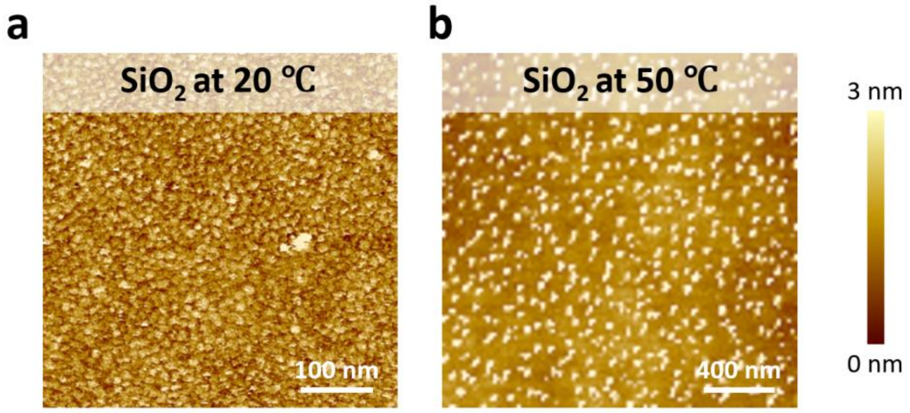

Figure S3. AFM images of Sb_2O_3 grown on SiO_2 substrates at different temperature. (a) 20 ℃. (b) 50 ℃.

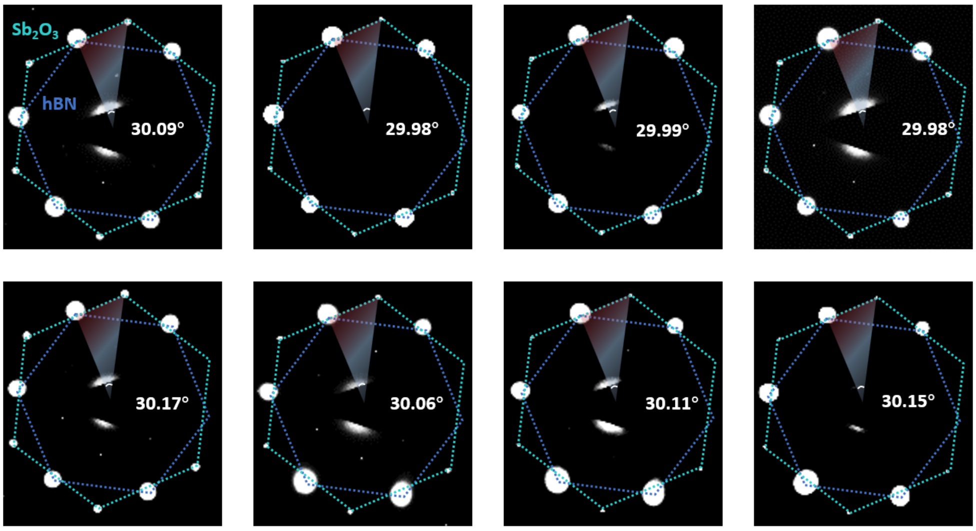

Figure S4. The original selected area electron diffraction (SAED) patterns of Sb_2O_3 deposited on hBN substrate.

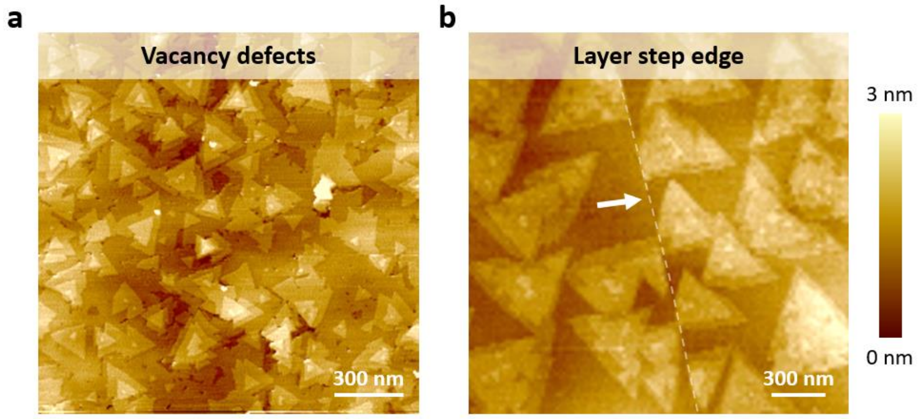

Figure S5. AFM images of Sb_2O_3 grown on substrates with defects. (a) Sb_2O_3 grown on graphene substrate with a large number of vacancies created through O_2 plasma treatment, demonstrating disarranged domains orientation. (b) Sb_2O_3 grown on HbN substrate with a layer step edge, which impedes the expansion of domains. The trace of the layer step edge is indicated by the dashed white line.

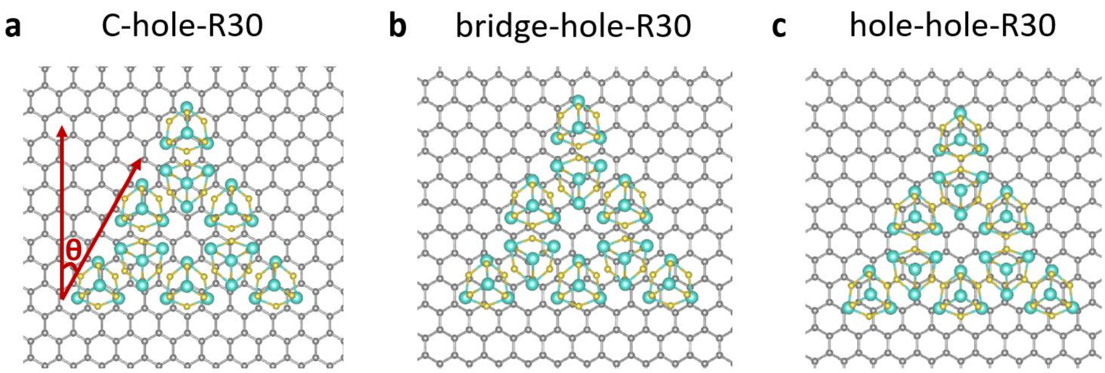

Figure S6. Configurations of Sb_2O_3 molecules adsorbed on graphene. The rotation angle is defined as the misorientation between the armchair direction of graphene and the edge of triangular Sb_2O_3 domains. For each rotation angle, 3 alignment styles were adopted, including (a) C-hole, (b) bridge-hole and (c) hole-hole.

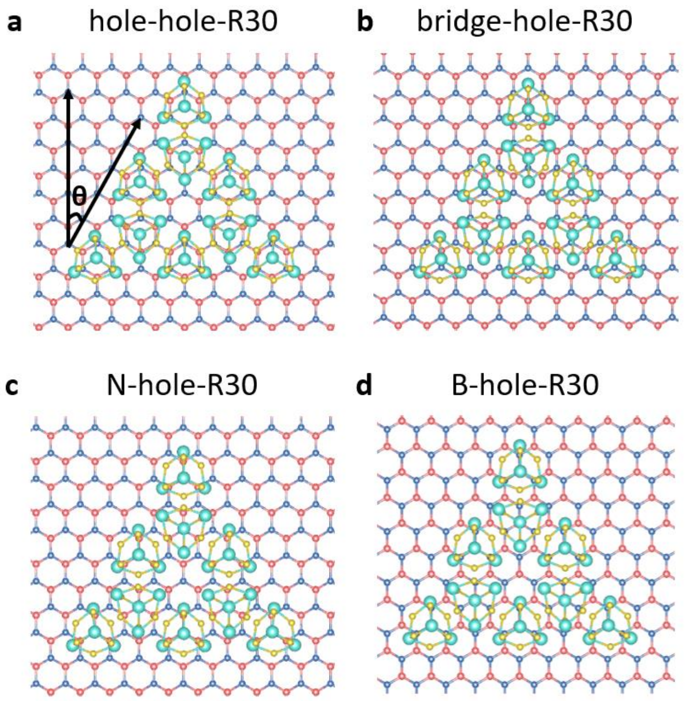

Figure S7. Configurations of Sb_2O_3 molecules adsorbed on hBN. For each rotation angle, 4 alignment styles were adopted, including (a) hole-hole, (b) bridge-hole, (c) N-hole and (d) B-hole.

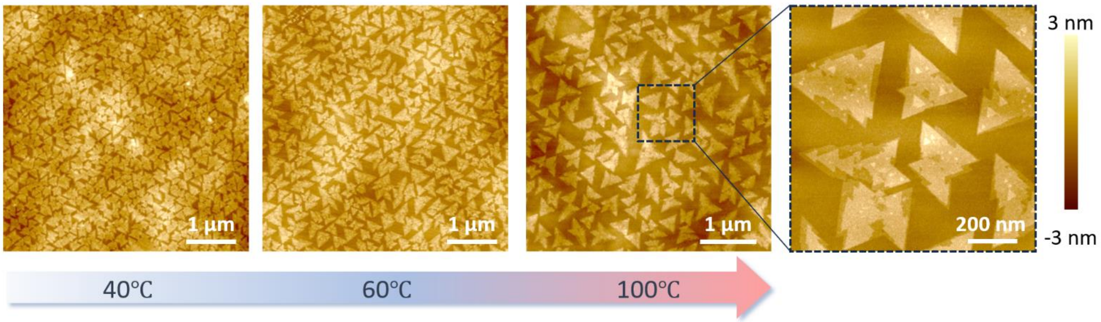

Figure S8. AFM images of Sb_2O_3 domains on graphene at substrate temperature of 40 ℃ to 100 ℃, demonstrating that temperature influents little on the orientation of Sb_2O_3 on graphene substrate, and the possibility for these two antiparallel domains emerging at different temperatures orientation remain equal.

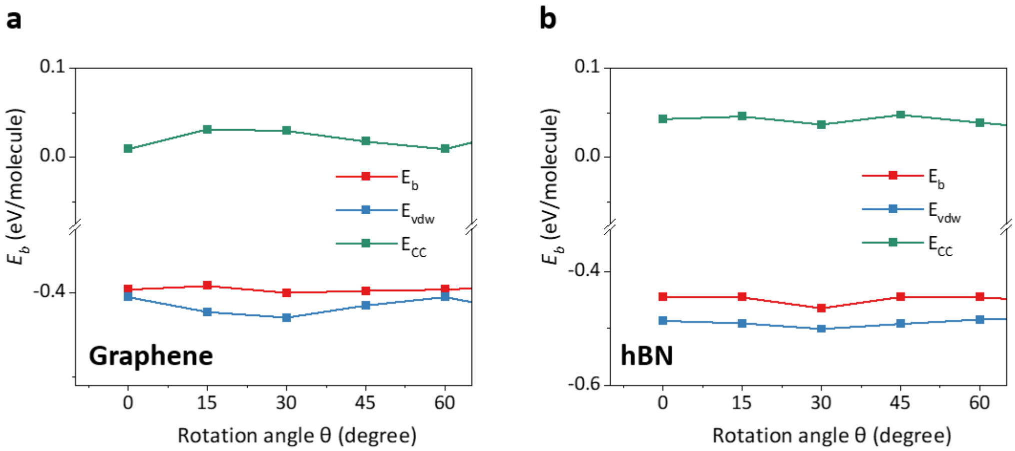

	E_b (eV/molecule)	E_{vdW} (eV/molecule)	E_{cc}(eV/molecule)
**hBN**	-0.464	-0.501	0.037
**graphene**	-0.400	-0.429	0.029

Figure S9. Decomposition of interlayer binding energy for Sb_2O_3 adsorbed on (a) graphene and (b) hBN. The results show that vdW interaction mainly contributes to the binding energy, and the contribution of other facts (mainly including electrostatic energy) to the binding energy can be ignored.

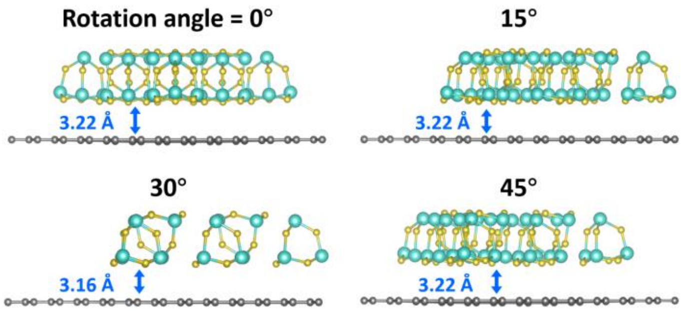

Figure S10. The equilibrium configurations of Sb_2O_3 nuclei on graphene substrate with different rotation angles.

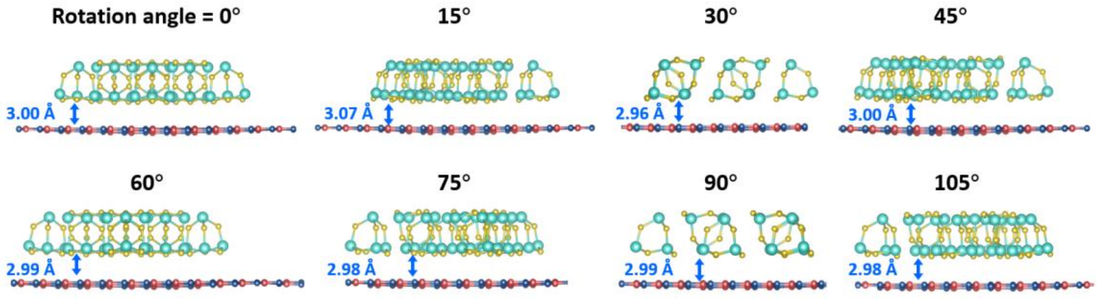

Figure S11. The equilibrium configurations of Sb_2O_3 nuclei on hBN substrate with different rotation angles.

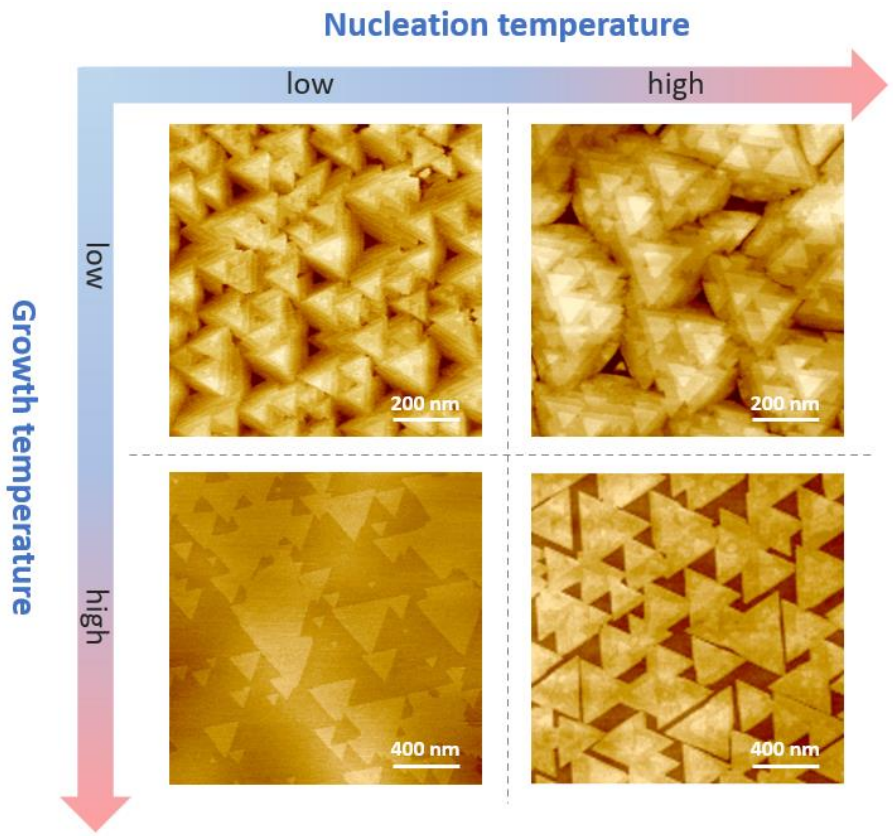

Figure S12. Effect of temperature on nucleation and growth stage.

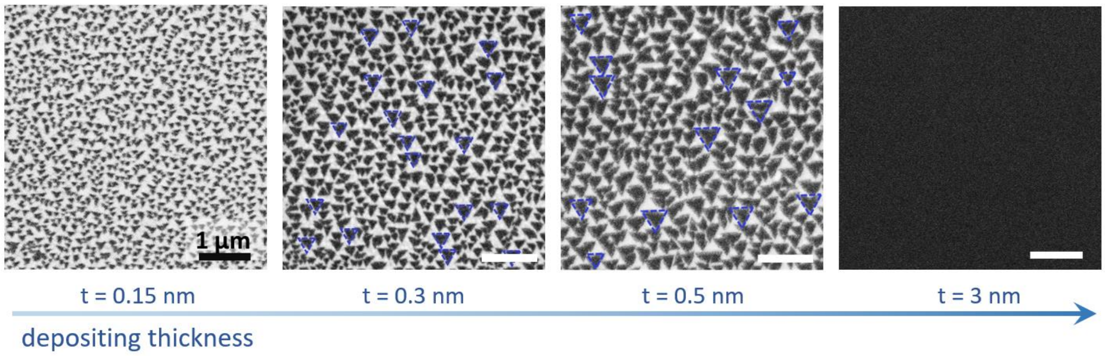

Figure S13. SEM images that demonstrating the growth process of Sb_2O_3 on hBN.

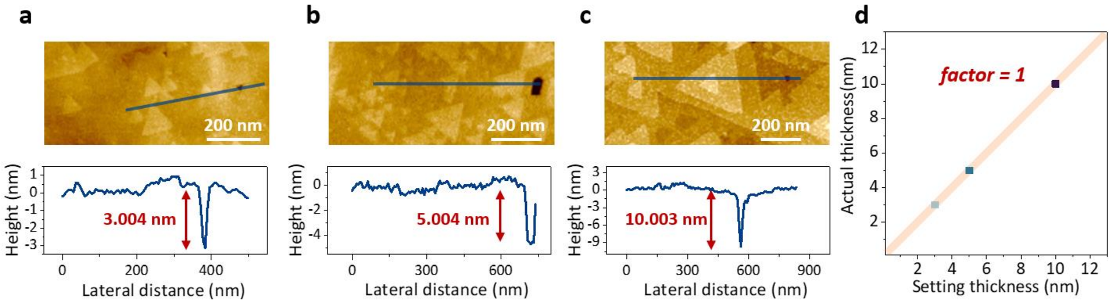

Figure S14. AFM images and corresponding profile curves of Sb_2O_3 films with different thicknesses. The hole is the uncovered area due to the defects in substrate. (a) 3 nm. (b) 5 nm. (c) 10 nm. (d) the relationship between setting thickness and the measured thickness.

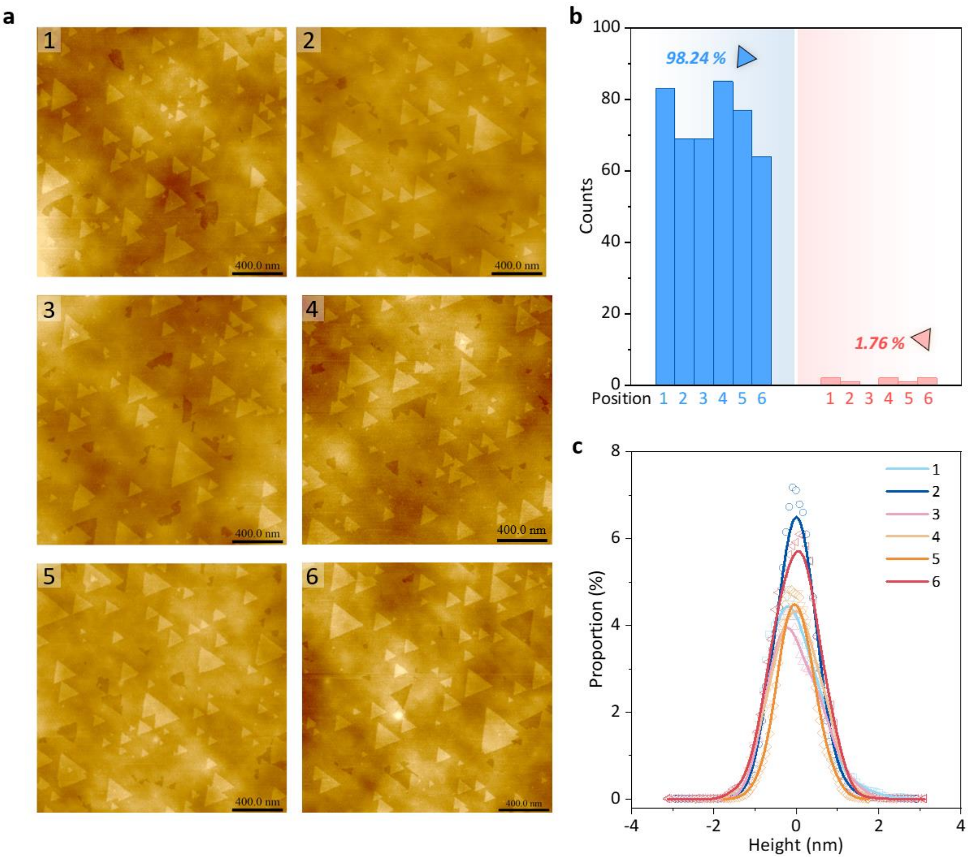

Figure S15. Orientation and thickness distribution of the single-crystal Sb_2O_3 film. (a) AFM images of the film surface from six random positions across a sub-millimeter-scale hBN substrate. (b) Statistics on the domains orientation of as-grown film according to the images in (a). (c) Height histograms for the surface of samples in (a).

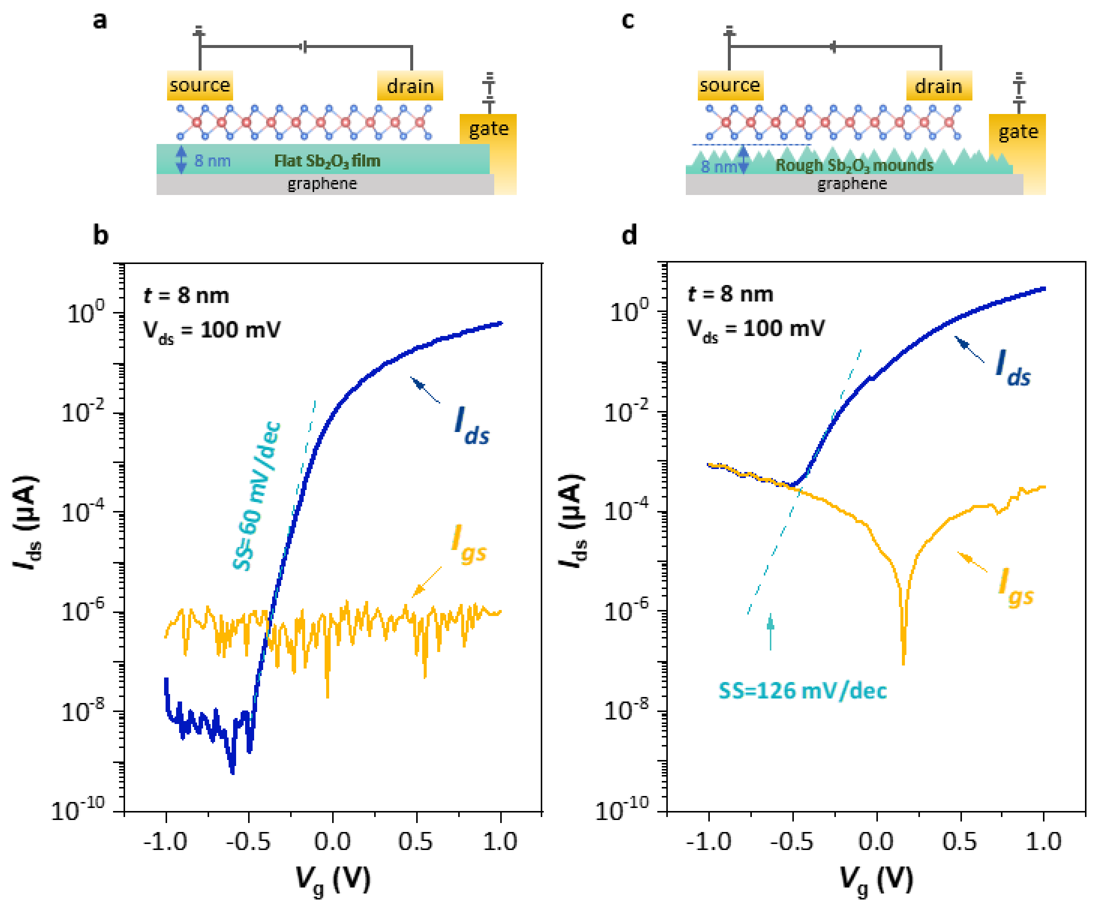

Figure S16. FETs based on the highly-oriented Sb_2O_3/graphene film. (a) Schematic and (b) Transfer characteristic curve of the device with flat Sb_2O_3 film as the dielectric. (c) Schematic and (d) Transfer characteristic curve of the device with rough Sb_2O_3 film as the dielectric.

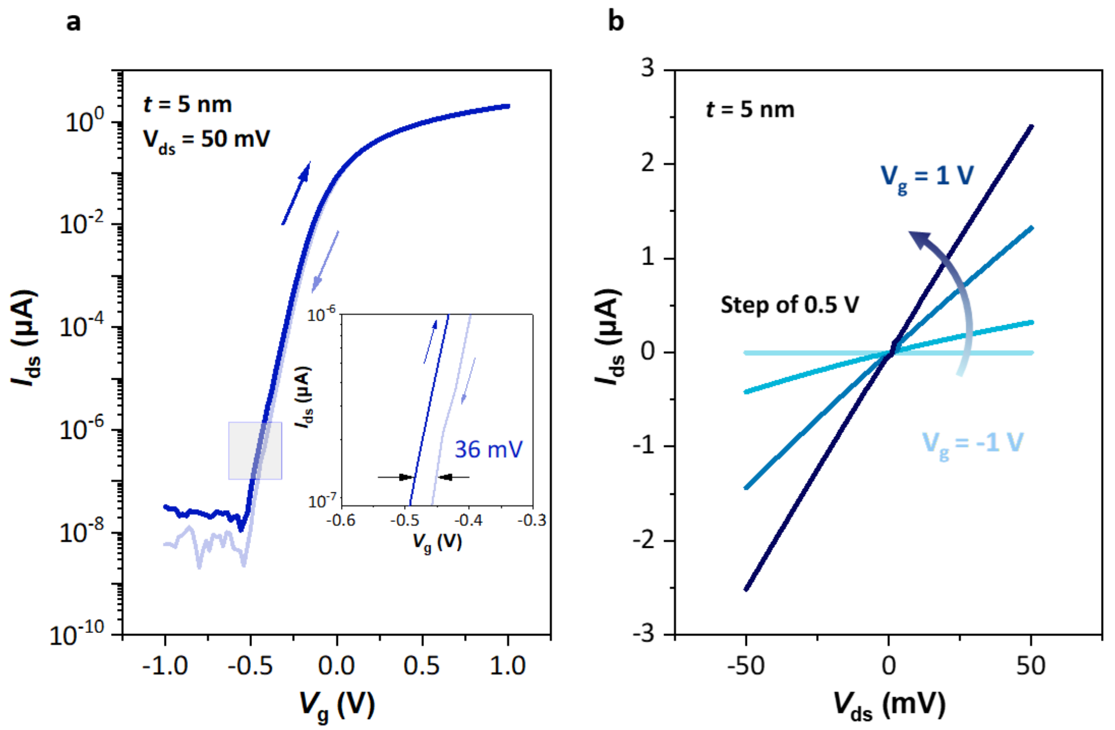

Figure S17. The electrical properties of MoS2-based FET with 5 nm highly-oriented Sb_2O_3 as the dielectric. (a) double-sweep transfer characteristic curves. (b) output characteristic curves.

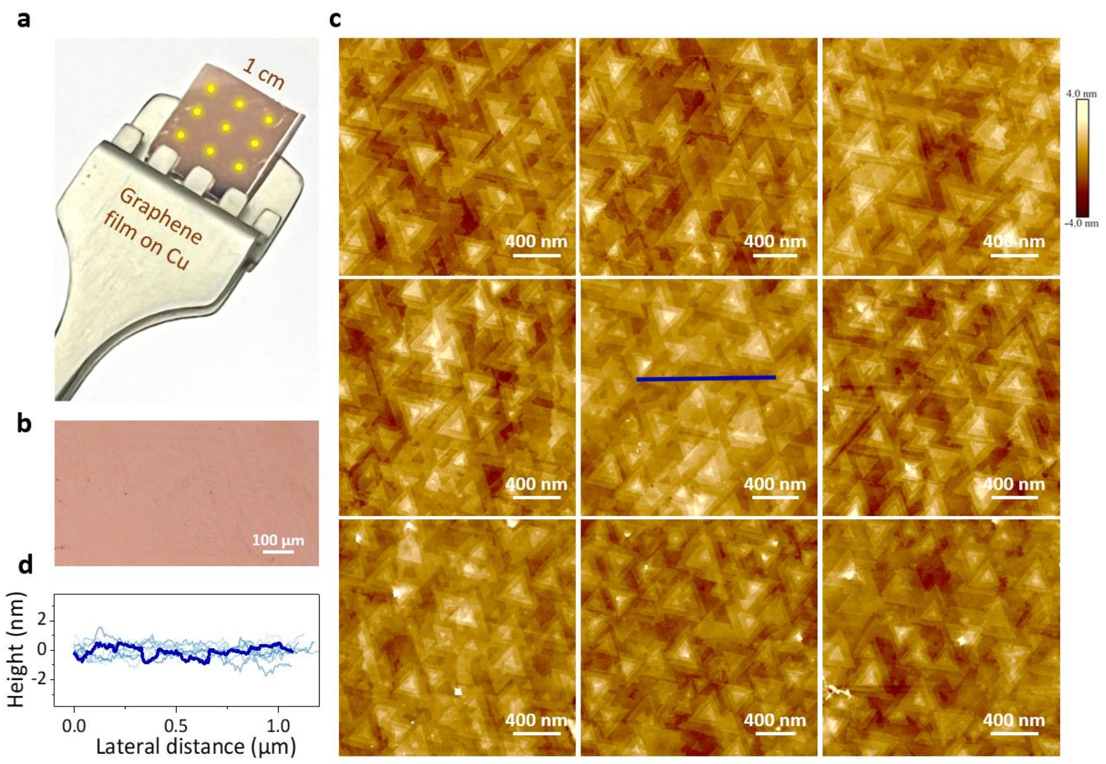

Figure S18. Centimeter-scale highly oriented Sb_2O_3/graphene film. (a) the photograph of the as-grown film. (b) OM image of the film. The blemishes can be attributed to the uneven surface of Cu foil beneath graphene. (c) AFM images of the Sb_2O_3 surface marked in (a). (d) Corresponding profile curves. From nine randomly selected sites over the whole film, the collected AFM images demonstrate that all of the domains were well aligned with two antiparallel directions and surfaces were atomically flat with only single-layer height fluctuation.

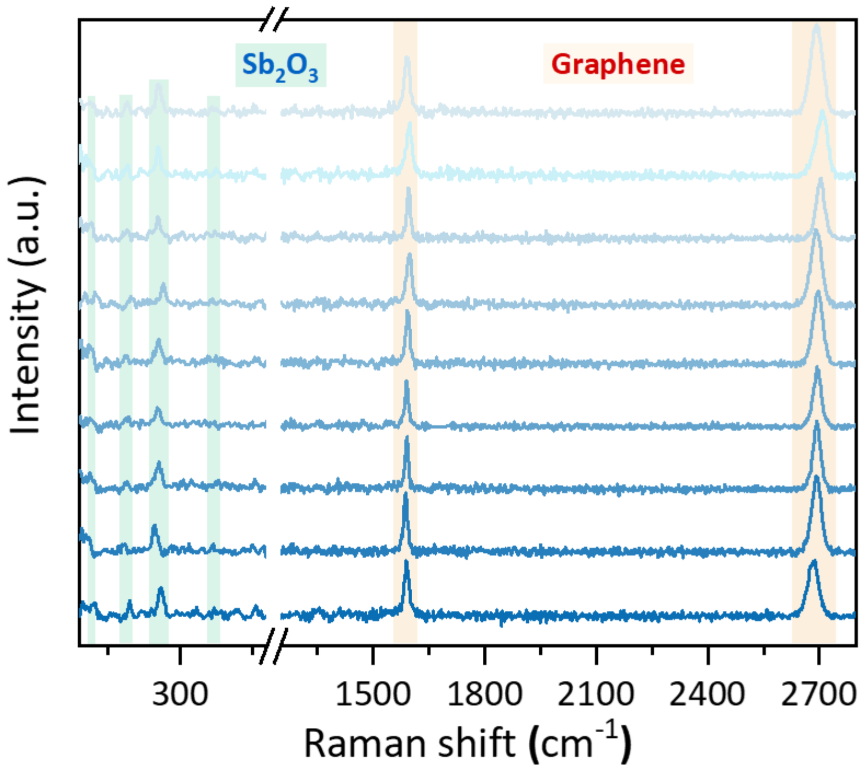

Figure S19. Raman spectra of the centimeter-scale highly oriented Sb_2O_3/graphene film at random positions. The spectra collected at the nine sites showed identical signal, revealing that the Sb_2O_3 film is uniform on the centimeter scale.

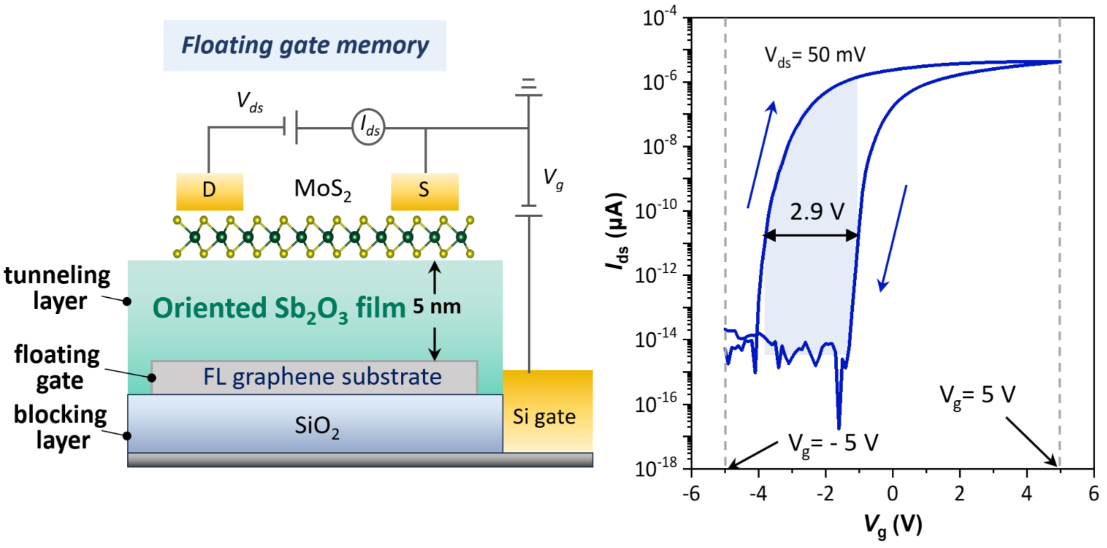

Figure S20. The device schematic and a double-sweep transfer characteristic curve of FGFET. The gate voltage was applied on Si, and graphene worked as the floating gate.

3. Supplementary Tables

Table S1. Statistical counting on orientations of Sb_2O_3 domains grown on graphene.

Growth temperature	Parallel counts ▲	Antiparallel counts ▼
**40 °C**	558	577
**60 °C**	457	462
**100 °C**	282	303

Table S2. Statistical counting on orientations of Sb_2O_3 domains grown on hBN.

Growth temperature	Parallel counts ▲	Antiparallel counts ▼
50 °C	2044	41
60 °C	2006	43
75 °C	1336	62
80 °C	603	61
100 °C	1363	314
110 °C	344	89

Table S3. Device performance comparison of MoS_2 FET gated by various dielectrics. [7-10]

Dielectric layer	EOT (nm)	SS (mV dec^{-1})	Leakage current (pA)	Operating voltage (V)	On/Off ratio	Ref.
**SiO_2**	300	1566 ± 460	NA	18	10^4	1
**HfO_2**	4.68	74	<2	1.55	10^6	2
**hBN**	11.7	NA	NA	10	10^4-10^6	3
**Polycrystalline Sb_2O_3**	3.3	64	10^{-2}	1	10^7	4
**Polycrystalline Sb_2O_3**	1.6	NA	200	0.5	10^4	4
**Highly-oriented Sb_2O_3**	1.6	60	10^{-2}	0.5	10^7	this work

Reference

1. Jia W, Cao Z, Wang L *et al.* The analysis of a plane wave pseudopotential density functional theory code on a GPU machine. *Comput Phys Commun* 2013; **184**: 9-18.

2. Jia W, Fu J, Cao Z *et al.* Fast plane wave density functional theory molecular dynamics calculations on multi-GPU machines. *J Comput Phys* 2013; **251**: 102-15.

3. Hamann DR. Optimized norm-conserving Vanderbilt pseudopotentials. *Phys Rev B* 2013; **88**: 085117.

4. Perdew JP, Burke K, Ernzerhof M. Generalized gradient approximation made simple. *Phys Rev Lett* 1996; **77**: 3865-8.

5. Grimme S, Antony J, Ehrlich S *et al.* A consistent and accurate ab initio parametrization of density functional dispersion correction (DFT-D) for the 94 elements H-Pu. *J Chem Phys* 2010; **132**: 154104.

6. Monkhorst HJ, Pack JD. Special points for Brillouin-zone integrations. *Phys Rev B* 1976; **13**: 5188-92.

7. Kalkan SB, Najafidehaghani E, Gan Z *et al.* High-performance monolayer MoS_2 field-effect transistors on cyclic olefin copolymer-passivated SiO_2 gate dielectric. *Adv Opt Mater* 2022; **11**: 2201653.

8. Radisavljevic B, Radenovic A, Brivio J *et al.* Single-layer MoS_2 transistors. *Nat Nanotechnol* 2011; **6**: 147-50.

9. Lee G-H, Yu Y-J, Cui X *et al.* Flexible and transparent MoS_2 field-effect transistors on hexagonal boron nitride-graphene heterostructures. *ACS Nano* 2013; **7**: 7931-6.

10. Liu K, Jin B, Han W *et al.* A wafer-scale van der Waals dielectric made from an inorganic molecular crystal film. *Nat Electron* 2021; **4**: 906-13.
